# Psychogenic Nonepileptic Seizures (PNES) in the Setting of Trauma and Schizophrenia

**DOI:** 10.1155/2023/6644876

**Published:** 2023-08-12

**Authors:** Sikander Chohan, Ali Chohan, Muhamid Asif

**Affiliations:** ^1^Wayne State University School of Medicine, Detroit, MI, USA; ^2^Western Michigan University Homer Stryker M.D. School of Medicine, Kalamazoo, MI, USA; ^3^ProMedica Physicians Family Medicine Fremont, Third Avenue Suite D, Fremont, OH, USA

## Abstract

Psychogenic nonepileptic seizures (PNES) are nonepileptic events characterized by seizure-like manifestations without abnormal electrical activity in the brain. Our case report illustrates the diagnostic journey of a young female with a history of schizophrenia and childhood trauma who had an initial misdiagnosis of epilepsy. The etiology of PNES is complex. Major depressive disorder and generalized anxiety disorder are common comorbid conditions in these patients. Additionally, previous trauma has been linked as a predisposing factor for the development of PNES. Psychotic disorders, specifically schizophrenia, have only recently been associated with PNES. We explore this relationship in depth, while also underscoring the diagnostic and treatment challenges of PNES that clinicians must remain aware of.

## 1. Introduction

Psychogenic nonepileptic seizures (PNES) are nonepileptic events in which patients experience altered states of consciousness, involuntary movements of limbs, impaired self-control, and brief episodes of unresponsiveness [1]. At surface-level, these events present identically to epileptic seizures. However, PNES is not associated with abnormal electrical activity of the brain on electroencephalogram (EEG) [1]. The exact pathogenesis of PNES remains a mystery; most clinicians suspect that the disorder has a psychological, rather than physiological, origin [2].

In the *Diagnostic and Statistical Manual of Mental Disorders*, *Fifth Edition* (*DSM-5*), PNES is categorized as a functional neurological disorder/conversion disorder. In the International Classification of Diseases for Mortality and Morbidity Statistics, 11th Revision (ICD-11), the disorder is categorized as “dissociative neurological symptom disorder, with non-epileptic seizures;” the presence of seizure-like disorders without a recognized disorder of the nervous system fits this diagnosis. Studies have demonstrated that PNES patients have a 15%–40% higher rate of past trauma and abuse compared to the control groups [2]. Similarly, concurrent mood and anxiety disorders are present in a majority of PNES patients [3]. These findings further support the theory that PNES may have a psychological origin.

Psychiatric comorbidities have long been associated with PNES [4]. Over 50% of patients with the disorder have such associations; this includes depression, anxiety, stress disorders, and personality disorders [4]. Greater psychiatric comorbidity burden is associated with poor outcomes in PNES [5]. Traumatic experiences have also long been associated with PNES [6, 7]. The association between psychotic disorders and PNES is less established.

In this report, we present the case of a young female with a past medical history of schizophrenia and childhood trauma who was diagnosed with PNES following an incorrect diagnosis of epilepsy. With this, we seek to further establish the relationship between PNES in the context of psychotic disorders. We aim to guide clinicians in diagnosing and treating PNES in such complex clinical situations. Informed consent has been obtained for the publication of this case report.

## 2. Case Presentation

### 2.1. Initial Visit

We present an 18-year-old female with a past medical history of pediatric autoimmune neuropsychiatric disorders associated with streptococcal infections (PANDAS), chronic insomnia, and schizophrenia who presented to the outpatient adult neurology office. Her childhood was notable for extensive verbal abuse and neglect from her mother, leading to untreated trauma. Three months prior to this visit, the patient was admitted to an inpatient psychiatric facility due to a 9-month history of persecutory delusions, auditory hallucinations, and blunted affect. She was diagnosed with paranoid-subtype schizophrenia and started on Haloperidol 5 mg twice a day. During her stay at the inpatient facility, she had multiple epileptic episodes; she was diagnosed with epilepsy and prescribed Lamotrigine. She initially started a dose of 25 mg once a day for 2 weeks and gradually increased to 50 mg once a day.

The patient scheduled the initial appointment for the chief concern of tremor. She described it as bilateral, occurring at rest and with action, and starting days after beginning Haloperidol. She described her schizophrenia as being well-controlled with the haloperidol and found the medication effective. Her vitals were within normal limits. Her physical exam was benign and only significant for akathisia and blunted affect. Specifically, on a neurological exam, she had intact cranial nerves I–XII, no focal sensory deficits, no motor strength deficits, and symmetric 2+/4+ deep tendon reflexes. Given the context, the tremor was likely an extrapyramidal side effect of Haloperidol. We consulted psychiatry, who recommended either decreasing the dose of haloperidol or switching to a second-generation atypical antipsychotic. The patient preferred to continue taking Haloperidol so we decreased her dose to 3 mg twice a day.

During the same visit, the patient expressed concern that her Lamotrogine dose was “not helping” her seizures. She described her seizures as starting at the age of eight. The seizures usually occurred after stressful situations involving the patient's mother. During the episodes, the patient loses motor control and falls to the ground with bilateral arm and limb shaking. She remains conscious during the episodes. Her uncle, present at the initial visit, described the episodes similarly and relayed that she kept her eyes closed and shook her head throughout the episodes. She did not bite her tongue, express incontinence, or have postictal confusion. Most lasted between “2–4 minutes.”

Even with the antiepileptic medication, the seizures continued to occur “every few weeks.” The patient was never treated for epilepsy before her inpatient stay 6 months ago. Since this visit focused on the patient's tremor, she was scheduled for a follow-up in a week regarding her seizures and told to continue to take the medications and call if she had another episode. She was also prescribed orders to draw routine labs.

### 2.2. Follow-Up Visits

At the time of the second appointment, the patient was found to have no abnormalities on complete blood count (CBC) and comprehensive metabolic panel (CMP). All hormones and inflammatory mediators were within normal limits. Her vital signs remained within normal limits. Physical exam remained unchanged from the last visit. Her tremor had noticeably improved as well.

The patient's seizures first started during childhood and centered around traumatic events involving her mother. This pattern, in combinated with the description that her conciousness remained intact during all episodes, pointed towards a diagnosis of PNES. The description of her seizures, specifically intact consciousness, further pointed toward this diagnosis. An EEG did not reveal abnormalities. Similarly, T1 and T2-weighted magnetic resonance imaging (MRI) of the brain was unremarkable. The patient was eventually admitted to the hospital to undergo video-EEG monitoring. Monitoring was done over a week, and on the last 2 days, the patient experienced multiple seizure episodes. EEG revealed no abnormal electrical activity during the events. Given the likely diagnosis of PNES, she was advised to gradually discontinue Lamotrigine over a period of weeks with close-monitoring. She also began cognitive behavioral therapy. The focus of this therapy was helping her recognize how her childhood trauma has impacted her and developing effective skills to combat the difficult situations. We specifically focused on her trauma with the aim of improving her quality of life, severity of schizophrenia, and treating her PNES.

## 3. Discussion

### 3.1. Psychogenic Nonepileptic Seizures Compared to Epileptic Seizures

One of the earliest proposed mechanisms of PNES revolved around the activation of dissociated material [8]. Traumatic events lead to a dissociation between one's consciousness and memories. In this view, PNES events are sensorimotor flashbacks that arise when debilitating memories temporarily reenter one's consciousness. Another model views PNES as a defense mechanism. Acute threats lead to hyperaroused states and seizure-like events occur to protect an individual from the physical and emotional toll of a possible threat [8]. Baselet [9] similarly proposes that PNES events may be akin to panic attacks; hyperarousal triggers a dissociative state characterized by emotional numbness, depersonalization-derealization, and seizure-like behavior. The patient may not be able to identify the trigger or even be aware of their dissociative response. A final model views PNES as habitual behaviors reinforced by the operant conditioning [8]. The seizure-like activity leads to an intrinsic or extrinsic benefit, and thus, are repeated continuously.

While many models predominantly view PNES as a physical manifestation of trauma and stress, the disorder likely has a multifactorial etiology. Brown and Reuber [8] propose that cognitive–emotional–behavioral disturbances in an individual's life lead to activation of a mental representation of seizures (known as the seizure scaffold). These disturbances may be related to anatomical arousal and dissociative states, but are not required to be so. Importantly, patients may activate the seizure scaffold without any consciousness awareness.

Due to its similar presentation to epilepsy, PNES can be difficult to diagnose. Up to 20% of patients diagnosed with epilepsy may actually have PNES [10]. Many patients even begin antiepileptic medications without resolution of seizures. Extensive evaluation, detailed patient history and physical examination, and neuroimaging studies are required in the workup of PNES. There are subtle differences between PNES and epilepsy that can help distinguish between the both disorders. Movements such as thrashing, pelvic thrusting, arched back, side-to-side head bobbing, and closed, clenched eyes suggest PNES. Partial responsiveness to stimuli and recall of the epileptic episode suggests PNES. Ictal dysautonomia and a lack of postictal confusion can also suggest PNES. However, a myriad of similarities still exist between PNES and epilepsy. Nearly a third of PNES patients report tongue-biting and over half report preictal auras [10, 11].

The gold-standard to differentiate between PENS and epilepsy is video-EEG monitoring. Nearly 70%–90% of patients will have a PNES episode within the first 48 hr of recording [10, 12, 13]. During the episode, video-EEG should show normal electrical patterns characteristic of an alert, awake patient. The video-EEG is usually preceded and followed up with awake-EEG's; if all three recordings are benign, PNES is the likely diagnosis.

Treating PNES is, in many aspects, just as challenging as diagnosing it. Clinicians must be thorough, and nuanced, in explaining how they came to this diagnosis. Initially, some patients may experience a reduction in PNES severity after learning of their diagnosis [14]. Patients first slowly discontinue use of antiepileptic medications. During this period, clinicians must closely monitor the patient for risk of new-onset epileptic seizures. Finally, all comorbid psychiatric conditions must be treated appropriately. Patients may benefit from cognitive behavior therapy, however limited evidence exists regarding its efficacy.

### 3.2. The Relationship between Psychogenic Nonepileptic Seizures and Psychotic Disorders

These are only a handful of reports of patients developing both schizophrenia and PNES. In 2007, Duncan et al. [15] and Devine and Duncan [16] described the onset of auditory hallucinations and abnormal motor movements leading to the development of PNES in a 23 year-old male. Another report describes a 42-year-old male with teenage-onset schizoaffective disorder and childhood abuse who presented to the emergency department with seizure-like episodes that were found to be PNES [17]. Most recently, a report by Banks and Plattes [18] in 2023 highlighted PNES in a 29-year-old male who developed schizophrenia at 23 years old.

Most of the patients in case reports describing schizophrenia and PNES report that a diagnosis of schizophrenia was made in adulthood. Then, years later a diagnoss of PNES was made. Both PNES and schizophrenia may result from exposure to extreme stress. In our patient, abuse from her mother likely led to undiagnosed PNES developing first. To our knowledge, this is the first report of PNES preceding development of schizophrenia. Both PNES and schizophrenia are associated withs severe traumatic experiences [7, 19]. Our patient did admit to severe stressors in her life, such as temporary homelessness and the death of family members, occuring in the year preceding the development of schizophrenia. The above-cited reports do not delve into details on their respective patients' schizophrenia diagnosis.

A study of 111 patients with schizophrenia and 85 control subjects determined that those with schizophrenia and childhood trauma had increased incidence of adult separation anxiety disorder [20]. Furthermore, the study found that schizophrenia in the setting of past childhood trauma is associated with increased disease burden [20]. This association itself may provide a key link between PNES, schizophrenia, and childhood trauma. Schizophrenia in the setting of untreated childhood trauma may lead to increased risk of development of numerous psychiatric disorders. Such patients will likely benefit from individualized therapy.

Limited research exists regarding whether concurrent schizophrenia and PNES lead to worse treatment outcomes or increased severity of disease. The studies show that schizophrenia in the setting of preexisting depression, anxiety, or PTSD is associated with worse outcomes [21]. PNES in the setting of preexisting depression, anxiety, or PTSD is also associated with worse outcomes [22]. However, in our patient, the dual diagnoses did not lead to the worse treatment outcomes; her schizophrenia remained well controlled with antipsychotics.

## 4. Conclusions

This report underscores the intricate diagnostic and therapeutic challenges encountered when dealing with PNES. When presented with a patient with seizure-like episodes, clinicians must obtain a thorough history and description of the events and possible PNES should be ruled out. Our patient's initial misdiagnosis further highlights the complexity of the disorder.

This report also establishes a possible association between PNES and psychotic disorders. Respectively, both disorders are known to arise, in part, due to extreme stressors. Whether one disorder predisposes patients to the other is unknown. Relatedly, it is unknown whether acute exacerbations of schizophrenia can lead to the development of PNES. Future studies must attempt to elucidate the relationship between PNES and psychotic disorders, as well as to establish the evidence-based therapeutic strategies tailored to this unique patient population. Future studies must also determine whether the coexistence of both disorders leads to worse treatment outcomes.

## Data Availability

Data availability is not applicable for this article.

## References

[1] Huff J. S., Murr N. (2022). Psychogenic nonepileptic seizures. *StatPearls*.

[2] Beghi M., Zhang L., Beghi E. (2020). History of violence/maltreatment and psychogenic non-epileptic seizures. *Seizure*.

[3] Erro R., Brigo F., Trinka E., Turri G., Edwards M. J., Tinazzi M. (2016). Psychogenic nonepileptic seizures and movement disorders: a comparative review. *Neurology: Clinical Practice*.

[4] Diprose W., Sundram F., Menkes D. B. (2016). Psychiatric comorbidity in psychogenic nonepileptic seizures compared with epilepsy. *Epilepsy & Behavior*.

[5] Reuber M. (2008). Psychogenic nonepileptic seizures: answers and questions. *Epilepsy & Behavior*.

[6] Bakvis P., Roelofs K., Kuyk J., Edelbroek P. M., Swinkels W. A. M., Spinhoven P. (2009). Trauma, stress, and preconscious threat processing in patients with psychogenic nonepileptic seizures. *Epilepsia*.

[7] Beghi M., Cornaggia I., Magaudda A., Perin C., Peroni F., Cornaggia C. M. (2015). Childhood trauma and psychogenic nonepileptic seizures: a review of findings with speculations on the underlying mechanisms. *Epilepsy & Behavior*.

[8] Brown R. J., Reuber M. (2016). Towards an integrative theory of psychogenic non-epileptic seizures (PNES). *Clinical Psychology Review*.

[9] Baslet G. (2011). Psychogenic non-epileptic seizures: a model of their pathogenic mechanism. *Seizure*.

[10] Benbadis S. R., Agrawal V., Tatum W. O. (2001). How many patients with psychogenic nonepileptic seizures also have epilepsy?. *Neurology*.

[11] Volbers B., Walther K., Kurzbuch K. (2022). Psychogenic nonepileptic seizures: clinical characteristics and outcome. *Brain and Behavior*.

[12] Asadi-Pooya A. A. (2017). Psychogenic nonepileptic seizures: a concise review. *Neurological Sciences*.

[13] Woollacott I. O. C., Scott C., Fish D. R., Smith S. M., Walker M. C. (2010). When do psychogenic nonepileptic seizures occur on a video/EEG telemetry unit?. *Epilepsy & Behavior*.

[14] Perrin M. W., Sahoo S. K., Goodkin H. P. (2010). Latency to first psychogenic nonepileptic seizure upon admission to inpatient EEG monitoring: evidence for semiological differences. *Epilepsy & Behavior*.

[15] Duncan R., Razvi S., Mulhern S. (2011). Newly presenting psychogenic nonepileptic seizures: incidence, population characteristics, and early outcome from a prospective audit of a first seizure clinic. *Epilepsy & Behavior*.

[16] Devine M. J., Duncan J. S. (2007). Development of psychogenic non-epileptic seizures in response to auditory hallucinations. *Seizure*.

[17] Tito E., Knapp B., Bucca A., Espiridion E. D. (2019). A case report of schizoaffective disorder with pseudoseizures in a 42-year-old male. *Cureus*.

[18] Banks E. M., Plattes M. M. (2023). A case report of psychogenic non-epileptic seizures in a 29-year-old male with schizophrenia. *Cureus*.

[19] Popovic D., Schmitt A., Kaurani L. (2019). Childhood trauma in schizophrenia: current findings and research perspectives. *Frontiers in Neuroscience*.

[20] Karaytuğ M. O., Tamam L., Demirkol M. E., Namlı Z., Gürbüz M., Yeşiloğlu C. (2023). Impact of childhood trauma and adult separation anxiety disorder on quality of life in individuals with schizophrenia. *Neuropsychiatric Disease and Treatment*.

[21] Abdullah H. M., Shahul H. A., Hwang M. Y., Ferrando S. (2020). Comorbidity in schizophrenia: conceptual issues and clinical management. *FOCUS*.

[22] Durrant J., Rickards H., Cavanna A. E. (2011). Prognosis and outcome predictors in psychogenic nonepileptic seizures. *Epilepsy Research and Treatment*.

